# The mechanism of action and therapeutic potential of tumor-associated macrophages in tumor immune evasion

**DOI:** 10.3389/fimmu.2025.1545928

**Published:** 2025-04-22

**Authors:** Kehua Wang, Xu Zhang, Aiqin Li, Xia Qiao, Yanan Xu

**Affiliations:** ^1^ Department of Vascular Surgery, General Hospital of Ningxia Medical University, Yinchuan, China; ^2^ Department of Surgery Laboratory, Institute of Medical Sciences, General Hospital of Ningxia Medical University, Yinchuan, China

**Keywords:** macrophage, tumor, TAMs, regulation of immunity, cytokines

## Abstract

Tumor-associated macrophages (TAMs) play a multifaceted role in tumor progression. As specialized immune cells, macrophages are capable of phagocytosis and digesting foreign substances, as well as removing harmful substances including cellular debris and tumor cells. Under specific pathological conditions, circulating monocytes can be recruited into the tumor microenvironment and differentiate into TAMs. Macrophages are generally polarized into two distinct subpopulations: classically activated macrophages (M1) and alternatively activated macrophages (M2). TAMs constitute a significant proportion of the mononuclear leukocyte population in solid tumors, exhibiting a complex and dualistic relationship with tumor cells. Substantial evidence indicates that TAMs can interact with tumor cells, facilitating their immune evasion while promoting invasion and metastasis. This review focuses on the mechanism and regulation of macrophages in the immune response to tumor cells, as well as various macrophage-based tumor-targeted therapeutic strategies. It will provide a reference for research on macrophage-centered therapy strategies and their application in clinical practice.

## Introduction

Macrophages play an important role in maintaining organismal homeostasis through their involvement in anti-infection defense, intracellular environmental stability, and host protection, primarily mediated by their phagocytic and digestive functions (1). Functionally, macrophages mediate both nonspecific defense mechanisms (innate immunity) and specific immune responses (adaptive immunity). Additionally, they exert an immunomodulatory role by secreting cytokines, the complement system, and inflammatory response regulation (2). Tumor cells, mesenchymal cells, and immune cells secrete chemokines and cytokines, and the monocytes in the blood are recruited into the tumor microenvironment and become tumor-related macrophages. TAMs are broadly classified into two distinct phenotypes: classically activated macrophages (M1) and alternately activated macrophages (M2) (3). These macrophage subtypes perform diverse functions in immune defense and surveillance, with their phenotypic plasticity allowing dynamic adaptation to microenvironmental changes (4). This review examines multiple mechanisms underlying macrophage-tumor cell interactions, with particular emphasis on TAM-mediated tumor promotion, immune evasion, and immunosuppression. Furthermore, we summarize recent advances in macrophage-targeted therapeutic strategies for tumor treatment.

## Biological characteristics and phenotypes of macrophages

Macrophages are multifunctional immune cells ubiquitously distributed in vertebrate tissues. Current evidence indicates that macrophages were mainly derived from peripheral blood monocytes (5). During the early stages of embryonic development, the monocytes recruited from the bone marrow to various tissues and organs through circulation, develop and differentiate into tissue-specific macrophages with specific functions (6). Multiple functionally distinct macrophage subsets have been described. According to the prevailing classification system, macrophages are polarized into two main phenotypes: classically activated (M1) and alternatively activated (M2) macrophages, a process mediated by cytokines secreted by CD4^+^ T helper (TH) cell subsets (7). Experimental studies have demonstrated that INF-γ, GM-CSF, and LPS could induce macrophage M1 polarization, which is characterized by the secretion of proinflammatory cytokines and elevated expression of major histocompatibility complex II (MHC II) (8). It is reported that M1 macrophages can enhance the activity of CD8^+^ T cells and NK cells in eliminating tumor cells or inducing tumor cell apoptosis by secreting cytokines such as TNF, IL-6, IL-12, and others (9). Conversely, alternatively activated M2 macrophages exhibit anti-inflammatory properties and participate in tissue repair processes. Indeed, Csf-1, IL-4, IL-13, and IL-10 could cause the polarization of macrophages into M2 macrophages (10). M2 macrophages are further classified into four subtypes based on their activation stimuli: M2a (induced by Th2 cytokines IL-4 and IL-13), M2b (activated by TLR ligands in the presence of immune complexes), M2c (polarized by IL-10), and M2d (the predominant phenotype in tumor microenvironment, commonly referred to as TAMs (11). It is important to note that M1 and M2 types only describe a simplified spectrum of macrophage polarization states. In contrast to M1 macrophages, M2 macrophages have a low expression of IL-12, a high expression of IL-10, and reduced antitumor activity (12).

**Figure 1:**
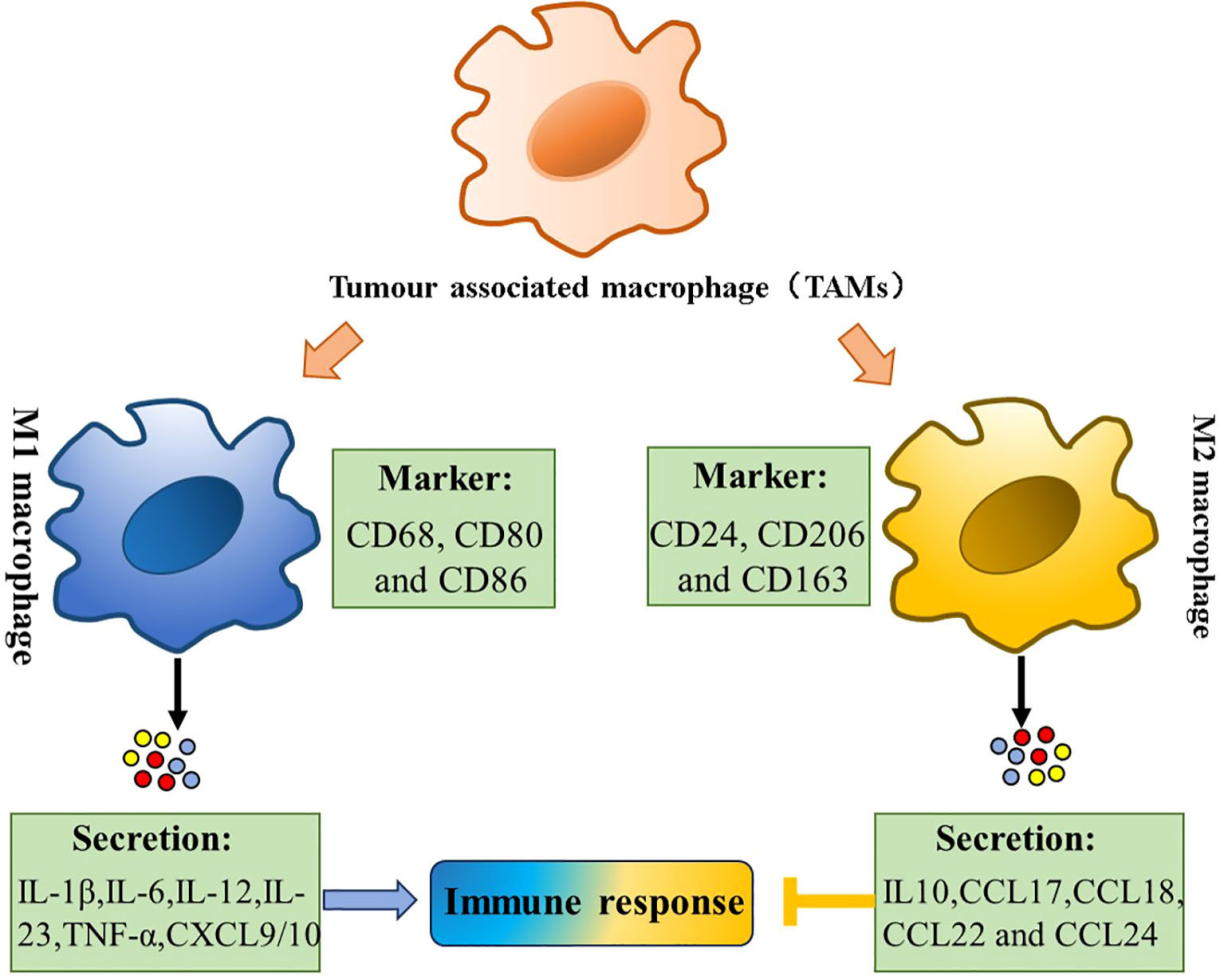
Tumor-associated macrophages (TAMs) polarization. Polarization of TAMs is regulated by multiple microenvironmental cytokines, and other signals derived. Two types of macrophages (M1/M2) secrete different immune markers, metabolic characteristics, and gene expression profiles to exert different functions.

Macrophages infiltrating tumor tissues or populated in the microenvironment of solid tumors are defined as TAMs (13). As a critical component of the tumor microenvironment, TAMs significantly influence multiple aspects of tumor biology, including tumor growth, tumor angiogenesis, immune regulation, metastasis, and chemoresistance (14). TAM interacts with a wide range of cell types, including fibroblasts, endothelial cells, various immune cells, and secreted factors (15). For example, CD4^+^ T cells polarize TAMs toward a pro-tumoral phenotype through IL-4, enhancing the TH2-type microenvironment. In contrast, in other cancers, this phenotype is stimulated by the complement component of humoral immunity (16). The phenotypic plasticity between M1 (anti-tumorigenesis) and M2 (pro-tumorigenesis) states represents a dynamic biological process that responds to microenvironmental signals (17). It is generally considered that M1 macrophages play an antitumor part in the early stages of tumor progression, which subsequently undergo phenotypic switching to M2-like macrophages that facilitate tumor progression (18) (**Figure 1**). TAMs primarily originate from monocytes that migrate to the tumor site. Upon entering the tumor microenvironment, they adopt various phenotypic states, often resembling M2-like or activated macrophages. These M2-like TAMs play crucial roles in tumor progression through multiple mechanisms, including angiogenesis promotion, metastasis facilitation, and immunosuppression within the tumor milieu.

## Role of macrophages in tumor growth

Macrophages are critical regulators of tissue homeostasis while simultaneously representing a prominent cellular component within the tumor microenvironment (TME). TME is a complex and dynamic milieu consisting of tumor cells and various stromal components, all of which interact and evolve in response to each other and therapeutic interventions (19). The number and phenotype of macrophages differed via tumor development (20). During the initial phases of tumorigenesis, macrophage infiltration demonstrates a substantial increase (21). Mounting evidence supports that inflammation is the root cause of many cancers (22). The role of inflammation in cancer initiation has direct experimental support. Unlike the wound healing and infections that resolve after immune cell recruitment and epithelial cell proliferation, growing tumors present with persisting oncogene-derived stress, cell death, and microbial signals that feed into a feed-forward loop of inflammation-induced signaling and inflammatory cell recruitment (23, 24). In a mouse model, the inflammation and tumorigenesis of lung cancer were increased after bronchial exposure to H. influenzae lysate (25). Chronic hepatitis, Helicobacter-induced gastritis, or Schistosoma-induced bladder inflammation increases cancer like colorectal cancer (CRC), liver cancer, and bladder cancer (26). Apart from bacterial infections, the inflammatory environment in the body caused by metabolic diseases was also important (27). During the advanced tumor stages, systemic inflammatory responses can activate neutrophils and induce extracellular trap formation, thereby facilitating breast cancer metastasis to pulmonary tissues (28). Another mechanism involves chemotherapy-induced intestinal tissue damage, which promotes microbial product translocation and systemic inflammation activation, ultimately accelerating metastatic progression (29) as demonstrated by most cancer-related mechanisms, where microbial products accelerate metastatic growth (30). The effects of inflammation on cancer development are complex and opposing. Acute inflammation responses typically exert antitumor effects, while chronic inflammation promotes it. Similar to how inactivation of the p53 pathway is commonly observed in neoplasia, the activation of the NFκB pathway is frequently seen in carcinogenesis, where it plays a crucial role in inflammation and cancer promotion (31).

## TAMs in tissue invasion and distant metastasis

Invasion and metastasis are hallmark features of cancer. In addition to the hematogenous and lymphatic metastatic pathways, cancer cells can also metastasize through peritoneal and perineural routes, which are typical for ovarian and pancreatic cancers, respectively. As ubiquitous and functionally critical components of the tumor microenvironment, macrophages play an important role in tumor biology (32). In established tumors, macrophages promote growth and dissemination to secondary sites. The role of TAMs in cancer cell spread and metastasis includes their involvement in the lungs, liver, brain, bones, peritoneal cavity and so on (33).

The lung is one of the most common sites for cancer metastasis in both patients and preclinical models. Cancer cells spread from the primary tumor via hematogenous or lymphatic routes or direct invasion (34). Macrophages promote metastatic processes through multifaceted mechanisms: (1) enhancing tumor cell motility, invasive capacity, and intravasation at primary sites; (2) facilitating extravasation, angiogenesis, and metastatic colonization at secondary sites; and (3) promoting immunosuppression through T-cell inhibition and conferring chemoresistance (32).

The liver, serving as a primary metastatic target organ particularly for colorectal and pancreatic carcinomas, possesses a distinctive immune microenvironment characterized by inherent tolerogenic properties. Macrophages contribute to hepatic metastatic niche formation by stimulating angiogenesis, cancer cell invasion, and extravasation, with liver macrophages producing hepatic growth factors that bind to c-Met on tumor cells, aiding their extravasation (33).

Bone metastases represent a frequent complication in advanced cancers. Macrophages play a vital role in maintaining bone homeostasis, and both bone-resident and monocyte-derived macrophages adopt protumoral roles in metastatic lesions, aiding cancer progression (35).

Brain metastases, constituting up to 90% of brain malignancies, are associated with poor prognosis due to the brain’s critical functions and limited regenerative capacity. Tissue-resident macrophages, such as microglia and border-associated macrophages, are abundant in the healthy brain. Microglia, essential for maintaining brain homeostasis, participate in synaptic pruning, injury response, blood-brain barrier modulation, and pathogen defense (35).

The heterogeneity of metastatic sites in cancer presents significant clinical challenges. Responses to conventional therapies, checkpoint blockade immunotherapy, and clinical manifestations can differ significantly in sites such as the brain, liver, peritoneal cavity, and bone, as seen in the resistance to immunotherapy in hepatic metastases. Accumulating evidence indicates that TAMs display distinct phenotypic and functional characteristics across various metastatic sites, reflecting organ-specific microenvironmental cues and tissue-intrinsic factors. Currently, due to inherent challenges and limitations, the use of clinically relevant mouse metastatic models and the systematic analysis of myeloid cell diversity in human tumor metastases may pave the way for diagnostic and therapeutic approaches targeting TAMs, which is crucial for addressing clinical challenges (36).

## Mechanism of inhibiting tumor progression based on TAMs

### Exploiting autophagy as a means of anticancer therapy

Programmed cell clearance mediated by macrophages plays an important role in tumor clearance and surveillance. Autophagy is an evolutionarily conserved cellular response that degrades cytoplasmic components through the lysosomal pathway (37). This fundamental cellular housekeeping mechanism is essential for maintaining cellular homeostasis and plays pivotal roles in cellular development and differentiation processes. Recent studies have shed light on the intricate relationship between autophagy and cancer, unveiling its profound influence on cancer development and response to therapy. However, the role of autophagy in cancer is far from static, characterized by modulation resulting from either dysregulation or hyperactivation of autophagic pathways in malignant cells (38).

Anticancer therapies simultaneously induce both apoptotic and autophagic pathways, with the latter typically serving as a protective mechanism against therapy-induced cellular stress responses. In such cases, the inhibition of autophagy can be a reasonable strategy to enhance the efficacy of anti-cancer therapies. A moderate level of autophagy protects the tumor from an unfavorable external environment and promotes its growth. Conversely, excessive autophagy levels trigger autophagic death of tumor cells, and many researchers have utilized this to induce apoptosis in tumor cells and achieved remarkable results. In tumor necrosis factor-related apoptosis-inducing ligand (TRAIL)-resistant lung cancers, candesartan, and gingerol are found to be effective in reducing the resistance by blocking autophagy flux and thus, have improved the treatment of TRAIL-resistant lung cancers (39). In hormone receptor-positive breast cancer, inhibiting autophagy to overcome endocrine resistance in estrogen receptor-positive tumors can enhance the anticancer effects of TAMs (40). Meanwhile, cancer cells are more autophagy-dependent than normal tissues. Clinical studies have demonstrated that Beclin-1, a crucial autophagy regulator, is frequently deleted in breast, ovarian, and prostate cancers, with its loss resulting in impaired autophagic activity and enhanced tumor cell proliferation (41). Bif-1 is another protein that regulates autophagy by interacting with Beclin-1, and its knockout inhibits autophagy, leading to an increase in cancer formation. Homozygous deletion of ATG5 causes liver cancer in a high-penetrance animal model. Experimental studies utilizing genetically engineered mouse models with ATG5 or ATG7 deletion have demonstrated significant suppression of tumor growth through autophagy inhibition. Thus, targeting autophagy directly is a therapeutic strategy for cancer therapy (42).

### Therapeutic opportunities to exploit apoptosis for cancer therapy

**Figure 2:**
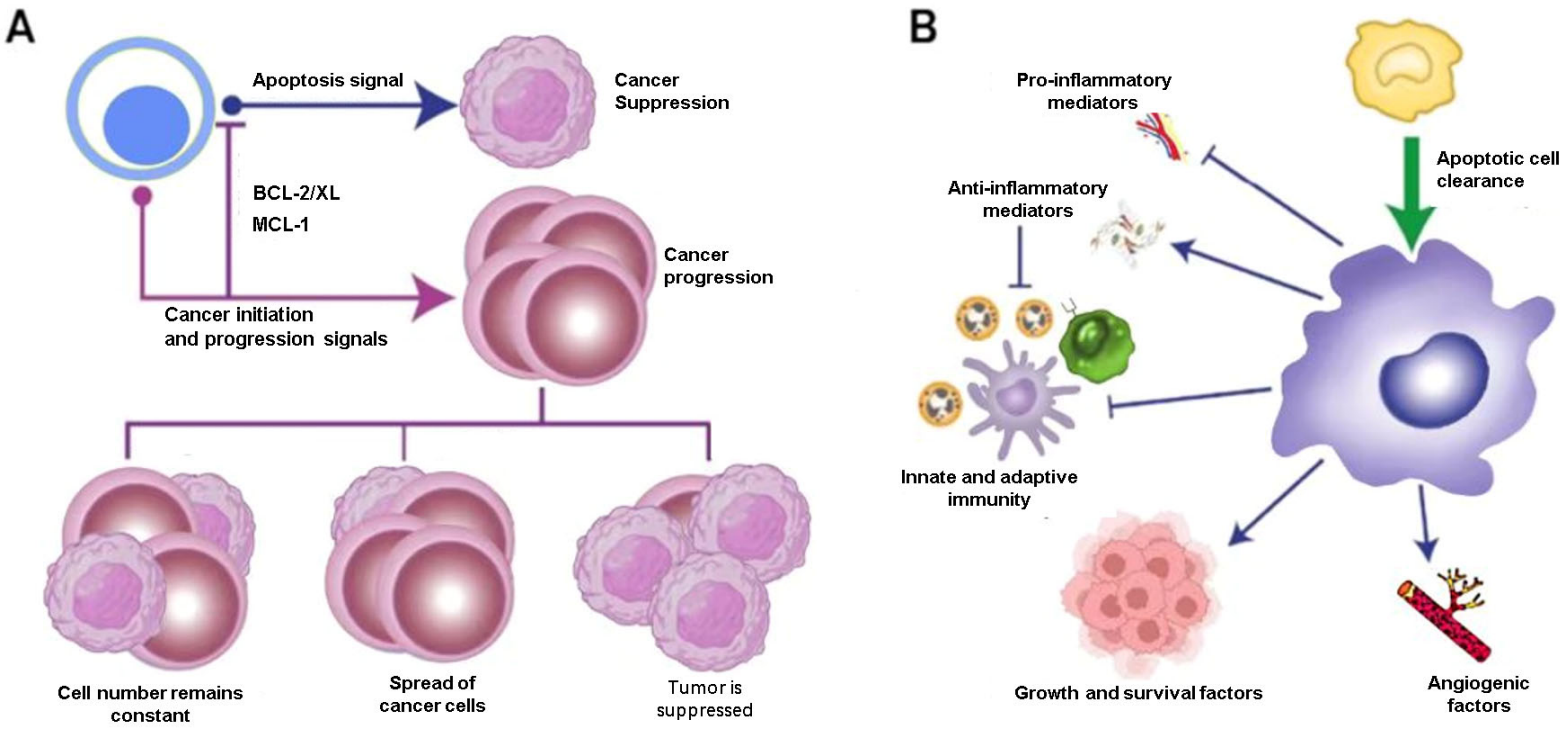
Therapeutic opportunities for cancer therapy. **(A)** Apoptosis is tumor-suppressive. For instance, some anti-apoptotic family members protect mutant cells from apoptosis, allowing the survival and outgrowth of premalignant or malignant populations. When the rate of cell death exceeds that of new cell generation, this balance is disrupted. **(B)** Phagocytic cells, particularly macrophages, play a role in this process and can exhibit pro-cancer characteristics. In some cases, apoptosis can also stimulate the body’s immune response.

Cancer growth represents a dysregulated imbalance between cell gain and cell loss, where the rate of increasing mutant tumor cells exceeds the rate of those that die. Apoptosis represents a tightly regulated and evolutionarily conserved cell death program, performing key functions in normal physiological processes such as embryogenesis and adult tissue homeostasis. The acquisition of apoptotic resistance represents a hallmark of malignant transformation, conferring survival advantages that facilitate tumor progression, evolution, and therapeutic resistance. In cancer, the balance between cell proliferation and apoptosis is disrupted, leading to uncontrolled growth and survival of malignant cells (43) (**Figure 2**). Notably, we present an overview of the implications of cell death programs in tumor biology, with a particular focus on apoptosis as a process with “double-edged” consequences: on the one hand, being tumor suppressive through deletion of malignant or pre-malignant cells, while, on the other, being tumor progressive through stimulation of reparatory and regenerative responses in the TME. Therefore, elucidating the molecular mechanisms underlying apoptotic regulation may unveil novel therapeutic opportunities for cancer management.

Several signaling pathways regulate apoptosis, including the intrinsic apoptosis pathway (the formation of the apoptosome and caspase 3 activation) and extrinsic apoptosis pathway (initiated by cell membrane proteins known as death receptors). Understanding these pathways and identifying specific targets within them have been instrumental in developing apoptosis-based cancer therapies. Bcl-2 family proteins were known to play an essential role in regulating the intrinsic pathway of apoptosis. The pro-apoptotic proteins Bax, Bak, Bad, and Bok could promote cytochrome c from the mitochondrial intermembrane space into the cytosol and cause induction of apoptosis eventually aiding in cancer therapeutics (43). Conversely, multidomain anti-apoptotic proteins (including Bcl-2, Bcl-xL, Bcl-w, Bfl-1, and Mcl-1) mediate apoptosis evasion in cancer cells and confer resistance to immune surveillance mechanisms (44). Currently, overexpression of BCL-2 has been clinically documented in acute myeloid leukemia, chronic lymphocytic leukemia (CLL), multiple myeloma, melanoma, hepatocellular carcinoma, lung cancer, breast cancer, and prostate cancer (45, 46).

Emerging evidence suggests that epigenetic mechanisms were usually regarded as involved in conferring apoptosis resistance. Epigenetic inactivation of critical apoptosis regulatory proteins could induce the transformation in key components of intracellular signal transduction (47). Engulfment of apoptotic cells is typically accompanied by activation of anti-inflammatory responses involving up-regulation of multiple factors, including TGF-β1, IL-10, PGE2, PGI2, and PAF. Concomitantly, primary pro-inflammatory mediators including TNF-α, IL-1β, IL-8, and IL-12 are downregulated. The molecular mechanisms underlying the recognition-phagocytosis-inflammation regulatory axis are gradually being elucidated. Some receptors, CD14, for example, are mainly involved in tethering apoptotic cells to phagocytes, whereas others, for instance, BAI1 and Stab2, clearly signal Rac-dependent phagocytosis and downstream lysosomal processing (48). A typical example of Caspase8, it was reported that Caspase8 was related to a variety of pediatric malignancies, including neuroblastoma, medulloblastoma, Ewing sarcoma, small-cell lung carcinoma, and has been linked to evasion of apoptosis (49, 50). These principles of pleiotropic responses to apoptosis by multiple different cell types in the TME, whether phagocytic or not, including healthy tumor cells, immune cells, fibroblasts, endothelial cells, and others, form the basis of a complex network of programs that help determine whether a tumor is benign, malignant, regressing or relapsing.

### Developed drugs that inhibit M2 polarization and enhance M1 activity in macrophages

**Figure 3:**
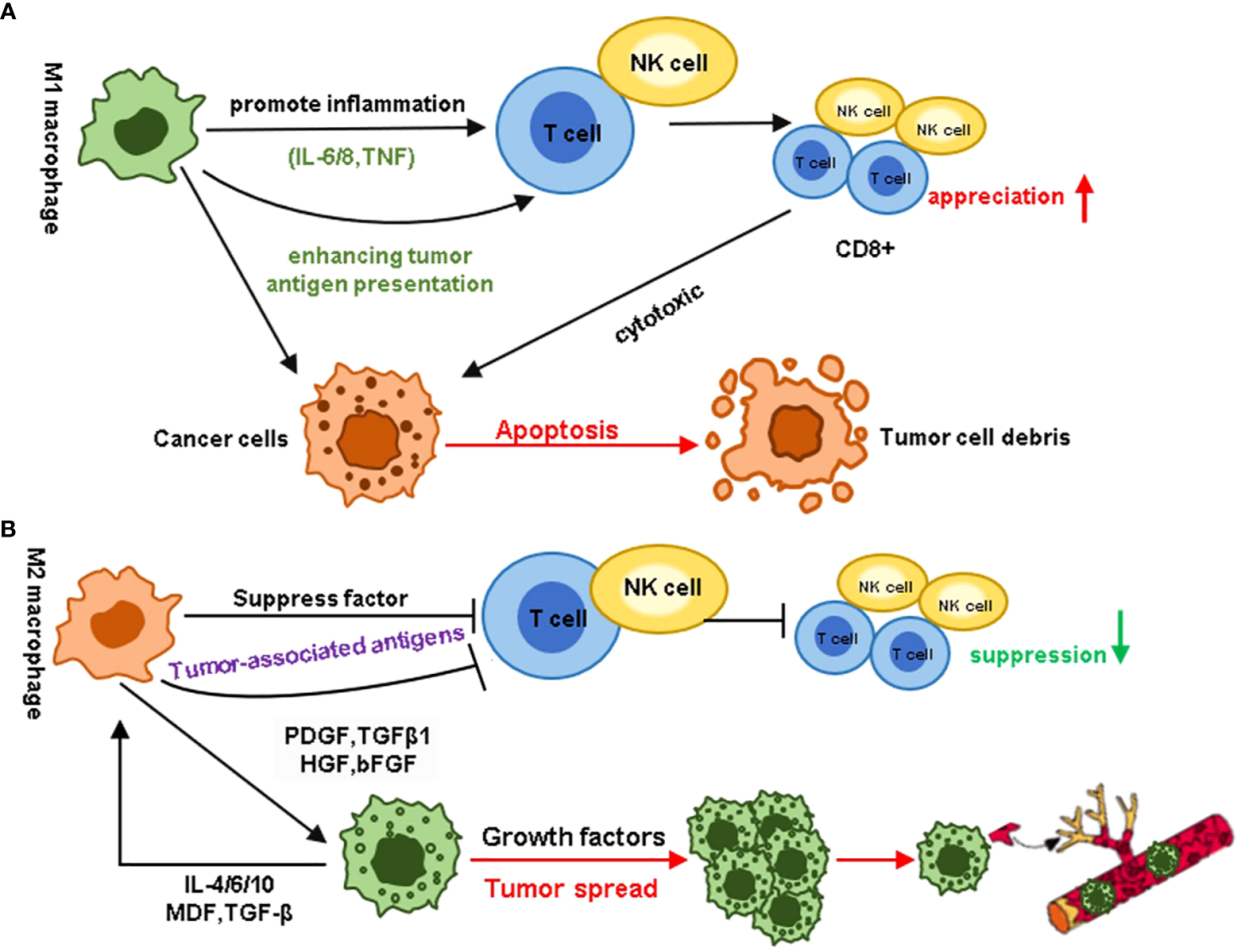
Different roles of MI and M2 macrophages in the tumor microenvironment. **(A)** M1 macrophages promote inflammation by enhancing tumor antigen presentation, stimulating the proliferation of immune cells such as CD8+ T cells and NK cells, and releasing pro-inflammatory cytokines like IL-6, IL-12, and TNF. In contrast, M2 macrophages promote tumor cell proliferation through immune suppression, tumor angiogenesis, new blood vessel formation, and the activation and remodeling of the extracellular matrix. **(B)** In contrast to the immune responses associated with M1 macrophages, M2 macrophages hinder the host’s immune status. In the tumor microenvironment, growth factors secreted by M2 macrophages can induce tumor cell proliferation and metastasis. Cytokines secreted by tumor cells, in turn, act as a feedback loop, further enhancing this effect.

M2 and M1 macrophages play opposing roles in tumor growth and metastasis. Therefore, inhibiting M2 macrophage polarization or converting the M2 phenotype to a tumor-killing M1 phenotype is a viable strategy for suppressing the role of TAMs in tumors. NF-κB, a key inducible transcription factor, regulates multiple signaling pathways involved in immune responses, inflammatory processes, cellular differentiation, and survival of both normal and malignant cells. Studies have confirmed that the activation of NF-κB is one of the key factors in inducing TAM differentiation towards the M1 phenotype. Previous research has found that Poria polysaccharides can bind to TLR4 proteins, activate NF-κB p65, and thereby regulate the polarization of M1 macrophages (51). Cordyceps extract can promote M1 macrophage polarization by activating the NF-κB pathway and inhibiting breast cancer growth (52). Currently, LPS and TLR receptors are important inducing factors that promote the development of TAM to M1. Previous studies revealed that the endothelial-mesenchymal transition-derived cancer-associated fibroblasts exhibited a potent tumor-promoting effect by secreting the heat shock protein 90α (HSP90α) and like to induce the M2 macrophage polarization. At the same time, TLR4 was the cell surface receptor for extracellular HSP90α (EHSP90α) (53). Numerous novel therapeutic agents have been developed with comparable macrophage-modulating effects. In recent years, scientists have developed novel types of drug-induced macrophages. Zoledronic acid (ZA), a therapeutic agent for treating skeletal-related events (SREs) and pain associated with bone metastasis. Studies have shown that ZA could reverse the polarity of TAMs from M2 to M1 by attenuating IL-10, VEGF, and MMP-9 production and recovering iNOS expression, moreover, ZA could prevent the number of macrophages in the TMEs (54). Huang et al. demonstrated that cationic polymer ethyleneimine (PEI) can alter the differentiation of TAM, promote the expression of IL-12, and reduce the content of IL-10 (55) (**Figure 3**). Histidine-rich glycoprotein (HRG), which contains abundant histidine residues (not histamine), can induce M2-to-M1 transformation, promote tumor vascular normalization, and enhance antitumor immunity (56).

## Mechanism of TAMs promoting tumor progression and immune escape

The immune system plays a critical role in recognizing and eliminating tumor cells through a highly complex process involving multiple immune components, among which macrophages serve as key participants. TAMs infiltrate the tumor stroma and play a crucial role in tumor progression and immune evasion. Investigating the interactions between TAMs and tumor progression lays the foundation for developing novel TAM-targeted therapeutic strategies and targeted treatments.

### Cytokines

The role of TAMs in promoting tumorigenesis and cancer progression has gained substantial recognition. In addition, TAMs can participate in tumor initiation by secreting signaling molecules and extracellular vesicles (EVs), providing structural support for tumor development. Extensive research has shown that TAMs secrete growth factors, cytokines, and chemokines that promote tumor progression, with significant advances made in understanding the roles of transforming growth factor-beta (TGF-β), IL-6, IL-8, and IL-10 in malignant tumor progression (57).

In colorectal cancer research, TAMs secrete transforming growth factor-beta (TGF-β), which promotes colorectal cancer proliferation and invasion by regulating the miR-34a/VEGF axis (58). Another mechanism involves TAMs promoting epithelial-to-mesenchymal transition (EMT) progression through the TGF-β/Smad2, 3-4/Snail signaling pathway (59). Inhibition of this pathway using TGF-β receptor inhibitors can suppress metastasis. Furthermore, TAM-secreted CXCL8 decreases ERα expression in endometrial cancer by modulating HOXB13, thereby promoting tumor progression. Lindsten et al. also found that TAMs are associated with poor prognosis in breast cancer patients (60). Abaurrea et al. discovered that IL-6 mediates EMT through the Janus kinase (JAK)/STAT3/Snail pathway (61). Another study showed that the combined activation of IL-6 and its receptor (IL-6R) induces STAT3 phosphorylation, which can resist apoptosis signaling pathways and promote tumor cell survival (62). IL-8 has emerged as a significant biomarker, with substantial evidence indicating its potential in predicting immune checkpoint inhibitor efficacy. Elevated IL-8 levels have been reported to promote tumor-invasive angiogenesis and immune suppression. Additionally, IL-8 promotes tumor metastasis by enhancing the expression of ELMO1 in tumor cells (63). IL-8 and chemokines are also upregulated in inflammatory breast cancer (IBC), where they recruit large numbers of monocytes, polarize macrophages, and promote tumor infiltration (64). The IL-10 cytokine family plays a dual role in maintaining tissue homeostasis during inflammatory responses while simultaneously influencing tumor progression. It has been reported that lipopolysaccharide (LPS) activation of TLR4 signaling significantly increases EMT in pancreatic cancer cells. Mechanistically, IL-10 enhances the expression of the cancer-associated phosphatase CIP2A through the PI3K signaling pathway, promoting lung adenocarcinoma invasion (65). Additionally, researchers have found that serum IL-10 levels correlate positively with tumor progression, suggesting that IL-10 plays an important role in tumor development (66).

### Immunosuppression

TAMs significantly contribute to tumor progression through immune suppression. As major immune regulatory cells within the tumor, TAMs have been extensively documented to inhibit cytotoxic T lymphocyte (CTL) responses in the tumor microenvironment, a finding that has been widely reported. Effective anti-tumor immunity largely depends on the activation of CD8^+^ T cells. However, TAMs, through multiple mechanisms, directly or indirectly inactivate CTLs, thereby facilitating tumor immune evasion and promoting tumor progression.

On the one hand, A variety of chemokines secreted by TAMs, cytokines (such as HGF, PDGF-B, VEGF, IL-4, IL-10), and enzymes (such as cathepsin K, cyclooxygenase-2, arginase 1 and matrix metalloproteinases) could inhibit directly the effects of CD8^+^T and CD4^+^T cells function. In addition, these chemokines, cytokines, and enzymes from TAMs stimulate the production of adaptive regulatory T cells (Tregs) and recruit natural regulatory T cells, Tregs (NTregs), to perform immunosuppressive functions either by directly inhibiting effector T cells or by secreting immunosuppressive factors (67). On the other hand, TAMs exert immunosuppressive effects through the expression of immune checkpoint molecules. For instance, TAMs isolated from hepatocellular carcinoma with high PD-L1 expression could inhibit tumor-specific T-cell immunity and promote tumor growth (68). In addition to directly on T cells, TAMs collaborate with other immune and stromal cells to establish an immunosuppressive microenvironment. Tregs and MDSCs are two other important immunosuppressive cell types in the tumor microenvironment. TAMs in glioma can recruit CCR2+Ly-6C+MDSCs and CCR4^+^Treg cells by producing abundant CCL2 (69).

### Exosomes of TAMs

Exosomes are nanoscale vesicles secreted actively by almost all cell types, including fibroblasts, endothelial cells, epithelial cells, neuronal cells, immune cells, and cancer cells. They mediate intercellular communication and material exchange, influencing the function of target cells. In malignancies, exosomes serve as pivotal mediators of material and information exchange within the tumor microenvironment, participating in various stages of cancer cell survival, growth, and metastasis. Their fundamental role in tumor biology has positioned exosomes as promising targets for cancer. Emerging evidence highlights the critical involvement of exosomes in mediating communication between cancer cells and immune cells (70). For instance, epithelial ovarian cancer (EOC)-derived exosomal miR-222-3p has been shown to reprogram macrophages into a tumor-associated phenotype through the SOCS3/STAT3 signaling pathway (71). Exosome-mediated transfer of functional CD11b/CD18 proteins from TAMs to tumor cells may enhance the migratory potential of hepatocellular carcinoma (HCC) cells (72). Exosomes collected from the liquid biopsy of acute myeloid leukemia (AML) patients’ liquid biopsies impair natural killer (NK) cell cytotoxicity through mechanisms involving increased Smad phosphorylation and downregulation of NKG2D receptor expression. These findings open a new avenue for studying the interactions between macrophages and tumor cells, shedding light on mechanisms that promote tumor progression and their potential clinical significance (73).

### Drug resistance to chemotherapy and radiotherapy

TAMs play two roles in chemotherapy drug therapy: improve the therapeutic effect or lead to chemotherapy resistance. Zitvogel. et al. early studies have suggested that macrophages exert host defense mechanisms that contribute to the therapeutic effect of adriamycin and stimulate anti-tumor immune responses (74). In addition, specific drugs, such as gemcitabine, have been reported to stimulate the cytotoxic potential and M1-like differentiation of macrophages. Among the negative effects of TAMs on chemotherapy, TAMs could reduce the efficacy of chemotherapy drugs through the following three mechanisms: 1) increase the recruitment of immunosuppressive myeloid cells; 2) Inhibition of adaptive anti-tumor immune response; 3) activation of anti-apoptotic programs in cancer cells. Preclinical studies have found that increased recruitment of macrophages is often observed in drug-resistant tumors, it is suggested that the mechanism of drug resistance is recruitment from TAMs (75). *In vitro* co-culture studies with macrophages and breast cancer cell lines have shown that macrophages contribute to chemotherapy resistance of paclitaxel, doxorubicin, and etoposide. *In vitro*, co-culture studies with macrophages and breast cancer cell lines have demonstrated that macrophages contribute to chemotherapy resistance of paclitaxel, doxorubicin, and etoposide. Specifically, compared with the control group, tumors treated with paclitaxel showed elevated CSF-1 mRNA expression in tumor cells after exposure to paclitaxel. Recruited TAMs inhibited paclitaxel-induced mitotic block, which leads to the development of drug resistance. Importantly, CSF-1/CSF-1 receptor (CSF-1R) signaling blockade has been shown to suppress TAM recruitment, enhance paclitaxel efficacy, and improve survival outcomes in murine models (76).

### Some other ways

Except for tumor cell-targeted therapeutic strategies, numerous strategies have been developed to target TAMs, such as CD40 agonists, phosphatidylinositol 3-kinase (PI3K)-γ inhibitors, and class IIa histone deacetylases (HDACs) inhibitors. CD40 is a member of the TNF receptor family, expressed on tumor cells, antigen-presenting cells (APCs), and macrophages. It has been reported that CD40 activation in macrophages can rapidly infiltrate tumors, kill tumor cells, and promote the depletion of the tumor stroma. Recent studies have shown that combining CSF-1R inhibitors with CD40 agonists reduces cell populations and promotes an inflammatory tumor microenvironment in murine colon cancer (77). PI3Kγ, the only member of the class-1B family, was expressed in bone marrow cells and macrophages. Activated PI3Kγ signaling could inhibit the activation of NF-κB and promote immune suppression during tumor growth (78). Moreover, in breast cancer animal models, PI3Kγ inhibition or deficiency rescues the immunosuppressive status by reprogramming the macrophages and inhibiting tumor cell metastasis and invasion (79). Class IIa HDAC inhibition also represents a novel therapeutic strategy for macrophage re-education toward anti-tumor phenotypes. Lorestani P et al. have indicated that the TMP195, a selective Class IIa HDAC inhibitor, induces the recruitment and differentiation of proinflammatory and phagocytic macrophages in the TME and repolarizes macrophages into an anti-tumor phenotype, reducing tumor burden and metastasis (80). Furthermore, combination therapy studies have shown that TMP195 combined with chemotherapy or checkpoint blockade treatment enhanced its tumoricidal effect in a mouse breast cancer model (80).

## Anti-tumor therapy targeting macrophages

TAMs represent a heterogeneous population of cells with diverse functions in homeostasis and pathological conditions. Given their characteristics, the study of TAMs has increasingly gained attention in cancer biology research. This functional diversity is tightly regulated by multiple microenvironmental factors and signaling pathways.

## Reprogramming the antibodies to TAM

Preclinical studies have shown that drugs that inhibit CD47 in cancer models can restore macrophages’ phagocytic and cytotoxic functions against tumor cells, leading to a robust anti-tumor immune response. Furthermore, combination therapies may enhance this efficacy. For example, combining an anti-SIRP-α blocking antibody and a CSF-1R inhibitor forms a supramolecular system that effectively stimulates the reprogramming of TAMs into the M1 phenotype (81). Moreover, De Sanctis F et al. have reported that PI3Kγ controls the switch from immune stimulation to suppression by inhibiting the NF-κB pathway and activating C/C/EBP-β, inhibition of PI3Kγ could promote pro-inflammatory cytokine expression, inhibition of tumor growth (82). In a mouse model of breast cancer, TMP195 administration induced the recruitment and differentiation of immunostimulatory CD40^+^ TAMs, resulting in tumor reduction. Moreover, the combination of TMP195 with chemotherapy regimens (carboplatin and paclitaxel) and immunotherapy (anti-PD1 antibodies) significantly enhanced the stability of the tumor response (83).

## Resistance of TAMs to cancer immunotherapy

Over the past decade, extensive preclinical studies and clinical trials have fully explored the efficacy and mechanisms of immunotherapy. Its ability to produce significant, lasting clinical responses has emerged as a breakthrough treatment for various refractory cancers, reshaping tumor treatment approaches. Chimeric antigen receptor T-cell immunotherapy has become a breakthrough treatment, with a success rate of over 90% for refractory B lymphocytic leukemia. Similarly, Immune checkpoint inhibitors (ICIs) have become cornerstone therapies, now established as first-line treatments for NSCLC, bladder cancer, melanoma, and renal cell carcinoma (84, 85).

Immunotherapy resistance can be classified as primary or acquired. Primary resistance, observed in up to 60% of certain cancer cases, is characterized by low clinical response rates. For instance, anti-PD-1/PD-L1 inhibitors can upregulate interferon-γ (IFN-γ), activating the JAK-STAT pathway, which leads to IRF8 expression and hyperprogressive disease (HPD) (86). Recently, Lin et al. identified that LRP1, a tumor promoter, inhibits DLL4 ubiquitination, activating NOTCH2 and driving epithelial–mesenchymal transition (EMT) and high CCL2 expression, which triggers M2-like macrophage polarization. These findings establish LRP1 as a promising therapeutic target in bladder cancer (87). Besides, polyadenylate-binding protein (PABPC1L) functions as a key factor in renal cell carcinoma immune evasion, enhancing indoleamine 2,3-dioxygenase 1(IDO1) and impeding T-cell function, and represents a potential target to enhance the efficacy of immune checkpoint blockade therapy (88). Acquired resistance occurs when a tumor initially responds to immunotherapy but relapses or progresses after a period of treatment. Adaptive immune resistance, a newly proposed mechanism distinct from traditional therapies, allows tumors to evade immune attacks by altering themselves to adapt to immune recognition (89). This type of resistance can arise through dynamic regulation of the immune microenvironment and interactions between immune and cancer cells, manifesting as primary resistance, mixed responses, or acquired resistance. The following section summarizes the specific molecular mechanisms through which TAMs contribute to immunotherapy resistance across various cancer types. (**Table 1**)

**Table 1:** The specific molecular mechanism of TAM affecting the development of cancer resistance to immunotherapy.

Mechanism category	Regulatory effect	Key molecules/signaling pathways
Immunosuppressive factor secretion	TAM secretes immunosuppressive factors and secretes IL-10 and TGF-β to inhibit the activity of T cells and NK cells and reduce the effect of immunotherapy.	IL-10, TGF-β
The expression of PD-L1 was up-regulated	TAM up-regulates PD-L1 expression in tumor cells through signaling pathways such as STAT3 and NF-KB, failing immune checkpoint inhibitors	PD-LIS AT3. NF-KB
Treg cell recruitment	TAM recruits regulatory T cells (Tregs) by secreting chemokines such as CCL22 to inhibit anti- tumor immune response further	CCL22 Tregs
Metabolic reprogramming	TAM inhibits T cell function and promotes tumor immune escape through metabolites such as lactic acid and arginase.	Lactic acid Arginase IDO

## Tumor metabolic alterations

Notably, macrophage polarization is associated with distinct metabolic profiles, which significantly influence the phenotype and functional characteristics of TAMs in cancer progression. Cancer cells can exploit metabolites to regulate tumor-infiltrating immune cells, promoting their differentiation and metastasis. For example, lactate secreted by glycolysis in cancer cells promotes the transfer of TAMs from pro-inflammatory (M1-like) to anti-inflammatory (M2-like) phenotypes. Another study has shown that membrane-cholesterol efflux drives TAM-mediated tumor progression. Ovarian cancer cells promote cholesterol efflux and increase IL-4-mediated signal transduction in TAMs, thus promoting tumor invasion and metastasis (90). By developing a predictive model, Lin H et al. demonstrated the impact of fatty acid metabolism on the efficacy of PD-1/PD-L1 inhibitors in clear cell renal cell carcinoma (ccRCC). These findings elucidate how fatty acid metabolism modulates the therapeutic response and to PD-1/PD-L1 blockade within the tumor microenvironment (TME) and its associated signaling pathways and M2-like macrophage polarization, highlighting the potential clinical applicability of this model (91).

## Macrophage polarization regulation

Manipulating the phenotype of TAMs represents a promising new approach for cancer immunotherapy. Several studies have shown that the macrophage phenotype is highly plastic and can be readily modulated by microenvironmental cues. This plasticity enables targeting of immunosuppressive TAMs, repolarizing them into pro-inflammatory cells that can effectively combat tumors and activate other immune system components (92). It is reported that many factors could stimulate immune responses and modulate TAM activity. Delivery of thymosin-α significantly activates TAM and transforms them into pro-inflammatory subsets that produce IL-1, TNF-α, ROS, and NO (93). This phenotypic conversion results in delayed tumor growth, and prolonged survival in mice. The role of microbial agents in TAM modulation has also been investigated. Thomas C J al. reported that using attenuated listeria to target TAM in the cancer microenvironment promoted the re-polarization of TAM into a pro-inflammatory phenotype (94). Besides, Drugs also play an important role in this process. Metformin, a well-known anti-diabetic drug, has demonstrated promising anti-cancer effects, although its underlying mechanisms remain incompletely understood. Further research showed that metformin may inhibit M2-like polarization of macrophages *in vitro* and *in vivo*, meanwhile, it could reduce the number of metastases in a murine model of Lewis lung carcinoma (95).

## The utilization of CAR-macrophages in cancer immunotherapy

While CAR T cell therapy has significantly progressed, its application in solid tumors remains underdeveloped. These obstacles include difficulties in CAR T cell manufacturing, lack of tumor-specific antigens, poor T cell trafficking, an immunosuppressive tumor microenvironment (TME), treatment toxicity, and antigen escape (96). Macrophages, abundant in the TME, efficiently infiltrate tumors and play a crucial role in immune regulation. Immunosuppressive M2 macrophages can phagocytose target cells similarly to proinflammatory M1 macrophages and can be reprogrammed to an M1 phenotype (97). This biological plasticity has stimulated considerable interest in developing CAR macrophage-based therapies as a promising alternative to address the limitations of CAR T cell therapy in solid tumors. However, CAR macrophages still have limitations that need to be addressed.

Target antigen selection is crucial in CAR T therapy. Ideal target antigens should exhibit tumor-specific expression patterns, being exclusively present on malignant cells. However, aside from cancer neoantigens and possibly EGFRvIII, most antigens used in CAR T therapy for solid tumors are shared by normal cells. Targeting tumor-associated antigens (TAAs) often leads to significant side effects due to the difficulty preventing off-tumor damage to normal cells in solid organs (98).

The chimeric antigen receptor (CAR) structure in CAR macrophages mirrors that of CAR T cells, consisting of an extracellular antigen-binding domain, hinge region, transmembrane domain, and intracellular domain. Klichinsky et al. developed and characterized CD3ζ-based anti-HER2 CAR macrophages using a replication-incompetent adenoviral vector for efficient and reproducible CAR delivery. Adenoviral infection induced M1 differentiation in CAR macrophages, shifting the TME from an anti-inflammatory M2 to a proinflammatory state. *In vivo*, these CAR macrophages significantly prolonged survival and reduced lung metastasis in mice with tumor implants (99). L. Zhang et al. addressed the inefficiency of bioengineering macrophages for cancer immunotherapy by using iPSC-derived CAR macrophages. Non-integrating episomal vectors were used to induce iPSC clones, which were then transduced with CAR containing CD86 and FcRγ intracellular domains. These CAR-iMACs initially exhibit an M2 phenotype but shift to a pro-inflammatory M1 state upon encountering target cancer cells, enabling them to engulf and attack tumors. *In vivo*, CAR-iMACs expanded, persisted, and demonstrated effective anti-tumor activity (100). Further advancements have been made in engineering human macrophages with enhanced tumor-targeting capabilities. Utilizing chimeric adenoviral vectors, researchers overcame the inherent resistance of primary human macrophages to genetic modification, generating CAR macrophages (CAR-Ms) with sustained M1 polarization. CAR macrophages (CAR-Ms) exhibited antigen-specific phagocytosis and tumor clearance *in vitro*. In two solid tumor xenograft models, a single infusion of CAR-Ms reduced tumor burden and prolonged survival. CAR-Ms expressed pro-inflammatory cytokines, converted bystander M2 macrophages to M1, upregulated antigen presentation, and activated T cells. Humanized mouse models further confirmed CAR-M-mediated TME remodeling and T cell activation (100). CAR macrophage is still at its nascent stage with only one clinical trial initiated and no results reported yet. Hence, many of the limitations have yet to be unfolded.

## Engineered macrophages

The success of nanocarriers in treating neurodegenerative diseases has inspired scientists to explore their potential for cancer therapy. *In vitro* studies have demonstrated that gold nanorods conjugated with macrolide sequences can accumulate in TAMs and exert a cytotoxic effect on surrounding cancer cells (101). This approach represents one of the most promising strategies for cancer treatment. Wang et al. used nanotechnology in the construction of an anticancer agent. To bind the magnetic shell of iron oxide to topoisomerase I inhibitor SN38 via carboxyl esterase joint and loaded it into RAW 264.7 macrophages. When these medicines are injected into the tumor site, SN38 is released and has anticancer effects. In addition, a similar macrophage-based combination therapy was designed. Constructed magnetic nanoparticles coated with mannose and loaded with 5-fluorouracil were applied in an intraperitoneal metastasis model of mice with breast cancer. Controlled release of 5-fluorouracil and tumor growth inhibition were observed when an electromagnetic field was applied in a mouse intraperitoneal metastatic model (102). (**Table 2**)

**Table 2:** Based on the methods described above, we have compiled a summary of the drugs developed to target macrophages, as shown in the table below:.

Drug Name	Target/Mechanism	Development Phase	Indications	Remark
CSFIR inhibitors				
Pexidartinib (PLX3397)	Inhibition of CSFIR reduces the number of TAMs	FAD approved (tenosynovial giant cell tumor)	Tenosynovial giant cell tumor, solid tumors (clinical trials)	hepatotoxicity
Emactuzumab (RG7155)	Anti-CSFIR monoclonal antibody, blocking the CSF1/CSFIR signaling pathway	Phase II clinical trial	Solid Tumors	Shows some antitumon activity but may cause fatigue and ederna
BLZ945	Selective CSFIR inhibitors reshape TAM phenotype	Phase I/II clinical trial	Solid Tumors	Combination with immune checkpoint inhibitors shows synergistic effects
CCL2/CCR2 antagonists				
Carlumab (CNTO888)	Anti-CCL2 monoclonal antibody blocks the CCL2/CCR2 axis and inhibits TAM recruitment	Phase II clinical trial	Solid Tumors	The efficacy of single drug is limited and needs to be combined with chemotherapy
PF-04136309	CCR2 antagonists inhibit monocyte recruitment into the tumor microenvironment	Phase II clinical trial	Pancreatic cancer	Combined with chemotherapy, it showed some anti-turnor activity
CD47/SIRPα targeted drugs				
Magrolimab(5F9)	Anti-CD47 monoclonal antibody blocks the “don’t eat me” signal and enhances macrophage phagocytosis of tumor cells	Phase Illelinical trial	Myelodysplastic syndrome, acute myeloid leukernia	May cause anemia, requiring step-by-step dosing
TTI/621(SIRPα-Fc)	SIRPα-Fc fusion protein, blocking CD47/SIRPα signaling	Phase I/II clinical trial	Hematological tumors solid tumors	Shows some anti-tumor activity
Other targets				
Trabectedin	Induce TAM apoptosis and reduce the number of TAMs	FAD approved (soft tissue sarcoma)	Soft tissue sarcoma ovarian cancer	May cause bone narrow suppression and hepatotoxicity
Maraviroe	CCR5 antagonists inhibit TAM recruitment and function	Phase II clinical trial	Colorectal cancer	Combination with immune checkpoint inhibitors shows synergistic effects
Seliciclib(R-roscovitine)	CDK inhibitors inhibit TAM tumor-promoting function	Phase II clinical trial	Non-small cell lung cancer	cause gastrointestinal reactions

## Investigation of the diverse subpopulations of TAMs within the TME

Current TAM-targeting therapeutic strategies demonstrate limited clinical efficacy, underscoring the critical need for improved treatment approaches to enhance cancer patient outcomes. Therefore, it is essential to further explore the heterogeneous subgroups of TAMs in the TME as potential targets. While TAMs-based therapy offers a promising approach to enhance anti-tumor immunity. However, the heterogeneity of TAMs and their complex cell-cell interactions make TAM-targeting strategies variable and unpredictable. This highlights the necessity of precisely characterizing distinct TAM subsets to optimize therapeutic strategies.

Single-cell RNA sequencing (scRNA-seq) is a powerful tool for analyzing tumor cell diversity and exploring TAM heterogeneity. For example, scRNA-seq has identified two TAM populations, C1QC^+^ and SPP1^+^, in colon cancer, with SPP1^+^ TAM depletion potentially improving myeloid-targeted immunotherapy (103). In chronic hepatitis B and C-related hepatocellular carcinoma (HCC), scRNA-seq revealed M2-like TAMs expressing CCL18 and CREM, which are enriched in advanced HCC and may contribute to tumor progression (104).

Much work remains to integrate single-cell resolution with clinical significance. These molecular findings into quantifiable clinical parameters will enable their integration into prognostic scoring systems, facilitating personalized treatment strategies and improving prognostic accuracy.

## Summary and outlook

This paper describes the origin, classification, and immune function of macrophages, and further explores the mechanism of macrophages’ involvement in the tumor microenvironment. Recently, TAM showed obvious heterogeneity and phenotypic plasticity, which is a major component of the microenvironment of solid tumors. The growing body of clinical and preclinical evidence underscores the therapeutic potential of targeting TAMs in anticancer strategies, with their inherent heterogeneity and distinct phenotypic characteristics providing a foundation for developing TAM-based personalized therapies (105). Current therapeutic approaches targeting TAMs focus on multiple strategies: (1) inhibiting monocyte recruitment to tumor sites. (2) selectively eliminating immunosuppressive M2 macrophages, (3) reprogramming immunosuppressive TAMs into antitumor phenotypes, (4) blocking M2-mediated tumor-promoting functions, (5) promoting M2-to-M1 phenotype conversion, and (6) enhancing macrophage-mediated tumor growth inhibition. These findings demonstrate that macrophages participate in tumor immune regulation through diverse molecular mechanisms, warranting further investigation and therapeutic exploitation.

With the development of precision medicine, the direction of tumor treatment has gradually shifted to precise targeted therapy. As tumor immunity has become a hot topic, lots of research has been used to overcome the bottleneck of traditional tumor therapy, but in recent years, this field has made limited progress in immunity. Concerning the complexity of TAMs, a greater understanding of interactions between macrophages and tumor cells is needed. More clinical data regarding the correlation between TAMs and patient outcomes are also needed to guide patient selection. Due to the limited efficacy of monotherapies, combinational approaches can address the shortcomings of TAM-targeted agents, conventional therapies, and other immunotherapeutics. Emerging evidence from preclinical studies and clinical trials indicates that TAM-targeted therapies can potentiate the efficacy of existing treatments (106). Future studies should focus on optimizing combination therapies to maximize immune activation while minimizing potential toxicities.

It has been studied that macrophages affect tumor cells through various mechanisms, and become a new focus and target of the immune system. In recent years, a variety of TAM recognition mechanisms have been discovered, and related targeted therapies, such as the application of monoclonal antibodies or inhibitors, gene modification, adoptive transfer of immune cells, and CAR-T are being further studied. Building on the success of CAR-T cell therapy, studies have developed CAR macrophages (CAR-M) for tumor immunotherapy. CAR-M therapy modifies macrophages with specific CARs to enhance phagocytosis and antigen presentation, converting M2 cells to the pro-inflammatory M1 phenotype. Compared to CAR-T, CAR-M offers unique advantages, including reduced non-tumor toxicity due to its limited circulation time. Currently, two FDA-approved clinical trials are investigating CAR-M therapy for HER2-overexpressing tumors and recurrent ovarian cancer or peritoneal mesothelioma using mRNA-targeted CAR-M approaches (107). CAR-M has shown significant benefits in solid tumors over macrophage reinfusion therapy. However, CAR-M development faces challenges in macrophage expansion and viral transduction efficiency.

Although targeting TAMs shows great potential in cancer therapy, its clinical application faces several challenges and potential side effects. TAMs play crucial roles in tissue repair and immune surveillance, so targeting them may cause off-target effects, such as tissue damage, immunosuppression, increased infection risk, and delayed wound healing. Additionally, TAMs are highly heterogeneous and plastic, with different subpopulations playing varying or even opposing roles in the tumor microenvironment, complicating the precise targeting of specific TAMs. Their dynamic changes and plasticity can also lead to treatment resistance, such as phenotype shifts or activation of alternative signaling pathways. Current TAM-directed therapeutic approaches, including CSF1/CSF1R inhibitors and CCL2/CCR2 antagonists, have demonstrated limited clinical efficacy as monotherapies, underscoring the necessity for combination strategies incorporating chemotherapy, radiotherapy, or immune checkpoint inhibitors (107). However, optimizing these combinations and addressing potential toxicity requires further investigation. Future research should focus on understanding TAM heterogeneity and regulatory mechanisms, developing more precise targeting strategies, and optimizing combination therapies for safer, more effective clinical application.

In conclusion, macrophages represent a promising therapeutic target in cancer treatment. While numerous therapeutic strategies have been developed, current technologies and clinical applications remain in their nascent stages with limited implementation. Consequently, numerous unexplored molecular mechanisms potentially play pivotal roles in regulating tumor progression, and several promising therapeutic targets warrant further investigation. Future research should prioritize elucidating the complex interplay and communication networks between macrophages and tumor cells to advance targeted cancer therapies.
